# Leptin action on nonneuronal cells in the CNS: potential clinical applications

**DOI:** 10.1111/j.1749-6632.2012.06472.x

**Published:** 2012-04-24

**Authors:** Weihong Pan, Hung Hsuchou, Bhavaani Jayaram, Reas S Khan, Eagle Yi-Kung Huang, Xiaojun Wu, Chu Chen, Abba J Kastin

**Affiliations:** 1 Blood-Brain Barrier Group, Pennington Biomedical Research CenterBaton Rouge, Lousiana; 2 Department of Pharmacology, National Defense Medical CenterTaipei, Taiwan, Republic of China; 3 Neuroscience Center of Excellence, Louisiana State University Health Science CenterNew Orleans, Lousiana

**Keywords:** leptin, CNS, obesity, astrocytes, blood–brain barrier

## Abstract

Leptin, an adipocyte-derived cytokine, crosses the blood–brain barrier to act on many regions of the central nervous system (CNS). It participates in the regulation of energy balance, inflammatory processes, immune regulation, synaptic formation, memory condensation, and neurotrophic activities. This review focuses on the newly identified actions of leptin on astrocytes. We first summarize the distribution of leptin receptors in the brain, with a focus on the hypothalamus, where the leptin receptor is known to mediate essential feeding suppression activities, and on the hippocampus, where leptin facilitates memory, reduces neurodegeneration, and plays a dual role in seizures. We will then discuss regulation of the nonneuronal leptin system in obesity. Its relationship with neuronal leptin signaling is illustrated by *in vitro* assays in primary astrocyte culture and by *in vivo* studies on mice after pretreatment with a glial metabolic inhibitor or after cell-specific deletion of intracellular signaling leptin receptors. Overall, the glial leptin system shows robust regulation and plays an essential role in obesity. Strategies to manipulate this nonneuronal leptin signaling may have major clinical impact.

## Introduction

Obesity is a global epidemic disorder. In the United States in 2010, no state had a prevalence of obesity less than 20%, and 12 states had a prevalence of 30% or more.^1^ Leptin is a key adipocytokine that plays an essential role in the regulation of obesity. It is a 16 kDa polypeptide mainly produced by adipocytes in fat tissue. Blood concentrations of leptin correlate with the amount of adipocity.^2^ Leptin levels are also increased in inflammatory situations. A product of the ob gene,^3^ leptin has many cellular targets in different organs. One of its major targets is the central nervous system (CNS). Leptin acts to reduce feeding behavior and inhibit obesity. After administration of recombinant leptin, it acts directly on neuronal networks that control feeding and energy balance.^4^

Leptin exerts its actions on the CNS via regionally expressed leptin receptors (ObR or LepR). Belonging to the cytokine receptor class I superfamily, the ObRs consist of five alternatively spliced variants: a, b, c, d, and e. Among them, ObRb has the longest cytoplasmic tail and is responsible for phosphorylation and activation of signal transducer and activator for transcription (STAT)-3. To exert its functions in most CNS regions, leptin must cross the blood–brain barrier (BBB), which it does by a unique transport system;^5^ ObRa is the most abundant isoform in the cerebral microvessels composing the BBB.^6^ Given sufficient levels of expression, as shown in cultured cells overexpressing ObR isoforms, all of the membrane-bound forms can efficiently endocytose leptin.^7^ By contrast, the soluble receptor ObRe may serve as an antagonist not only to leptin signaling but also to its transport across the BBB.^8^ In this review, we focus on nonneuronal cellular effects of leptin in the CNS and address pathophysiological implications of these findings, not only in obesity but also in several CNS disorders. This extends beyond studies of the transport of leptin across the BBB.^5^

## ObR distribution in the brain

In the initial study with nonradioactive *in situ* hybridization (ISH) of ObR mRNA in the brain of C57 mice, Huang *et al.* showed that the areas with the highest level of expression include the arcuate nucleus and median eminence of the hypothalamus. The dentate gyrus and CA1 region of the hippocampus are the second most abundant. Low levels are also seen in the piriform cortex and the medial margin of the medial habenular nucleus. Most of the ISH signals appear to be present in neurons, such as those producing neuropeptide Y.^9^ This was confirmed by an independent study, which also showed the presence of ObR mRNA in the choroid plexus.^10^ In the obese ob/ob mouse devoid of leptin production, ObR mRNA is increased in these hypothalamic regions, piriform and olfactory cortices, and the medial habenular nucleus in comparison with lean mice.^11^ Although the control conditions may not be ideal, it appears that upregulation of ObR has regional specificity and may be dependent on the inducing factors, such as obesity, fasting, ischemia, or inflammation.

ISH with isoform-specific probes showed that ObRa and ObRb are abundant in the hypothalamus, whereas ObRa, ObRc, and ObRf, but not ObRb, are prominent in the choroid plexus. Besides brain regions described in the preceding paragraph, the thalamus, substantia nigra, and granular cell layer of the cerebellum also show intense ObR signals.^12^ When relative levels of ObR isoforms are compared with those measured by reverse transcriptase–polymerase chain reaction (RT-PCR), the ObRb to ObRa mRNA ratio is highest in the hypothalamus of the normal adult mouse brain.^13^ Shioda *et al.* first showed the protein expression of ObR by immunohistochemistry (IHC) and western blotting (WB) in rat brain. ObR immunoreactivity was seen in the arcuate, paraventricular, and ventromedial nuclei of the hypothalamus, lateral hypothalamus, olfactory bulb, neocortex, cerebellar cortex, dorsal raphe nucleus, inferior olive, nucleus of the solitary tract, and dorsal motor nucleus of the vagus nerve, with WB showing a molecular weight corresponding to the 120 kDa major band.^14^ The levels of protein and mRNA expression are congruous. In human autopsy samples, ObR immunoreactivity is seen in choroid plexus epithelium, ependymal lining, neurons of the hypothalamic nuclei (arcuate, suprachiasmatic, mamillary, paraventricular, dorsomedial, supraoptic, and posterior), nucleus basalis of Meynert, inferior olivary nuclei, and cerebellar Purkinje cells. The molecular weights are 97 kDa and 125 kDa. However, there was no significant regulatory change by obesity and diabetes in comparison with lean subjects.^15^ More recently, advances in transgenic technology enabled a novel approach to generate a LepRb-IRES-Cre EYFP reporter mouse line. These mice have high levels of ObRb mRNA/EYFP coexpression, including in areas previously not shown to have abundant ObR, such as the dorsomedial nucleus of the hypothalamus, ventral premammillary nucleus, ventral tegmental area, parabrachial nucleus, the dorsal vagal complex, insular cortex, lateral septal nucleus, medial preoptic area, rostral linear nucleus, the Edinger–Westphal nucleus, and midbrain. The transgenic mice are thus useful to locate ObRb^+^ cells and study their regulatory changes.^16^

**Figure 1:**
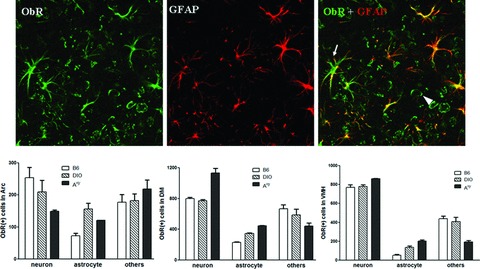
Obesity changes the number of ObR^+^ cell types in hypothalamic regions. Upper panel: some ObR^+^ cells are also GFAP^+^, shown by confocal images in the arcuate nucleus of a B6 male mouse. Lower panel: counting of demarcated regions from independent sections from four mice showed differential changes in the number of ObR^+^ neurons, astrocytes, and other types of cells in the arcuate nucleus (ArC), dorsomedial hypothalamus (DM), and ventromedial hypothalamus (VMH) of DIO and A^vy^ mice.

With regard to the cellular distribution of ObR in the brain, choroid plexus epithelia show a heterogeneous distribution of strong immunoreactivity. This population is probably responsible for leptin transport across the blood–cerebrospinal fluid barrier.^17^,^18^ ObR mRNA is also seen in meninges and blood vessels.^19^ Among CNS parenchymal cells, neurons are the most studied target; indeed, leptin-induced immediate early gene c-Fos activation mainly resides in neurons, as seen in a study focusing on hypothalamus and brainstem.^20^ However, ObR mRNA is also seen in astrocytes, as fluorescent ISH with double-labeling immunostaining of glial fibrillary acidic protein (GFAP) may be colocalized in normal rat hypothalamus.^21^ In the arcuate nucleus of the hypothalamus, about 20% of ObR immunoreactivity is seen in GFAP^+^ astrocytes, and this is further increased in mice with adult-onset obesity (Fig. 1, modified from Ref. 22), as will be further discussed in this review. Young showed that astrocytes have a specific anatomical relationship with leptin-sensitive neurons.^23^ Later, the distribution and upregulation of astrocytic ObR was shown in mouse models of adult onset obesity.^22^,^24^,^25^ Leptin can attenuate oligodendrocyte development embryonically,^26^ promote neurosphere self-renewal,^27^,^28^ increase angiogenesis after stroke,^28^ and show robust effects on microglial cytokine production.^29^–^31^

## Extrahypothalamic actions of leptin in the brain

Besides its effect on neuroendorine regulation of feeding, the extrahypothalamic actions of leptin are becoming increasingly recognized. One major site of action is the hippocampus.^32^,^33^ Leptin activates large conductance Ca^2+^-activated K^+^ channels in the hippocampus and promotes long-term depression of excitatory synaptic transmission.^34^–^38^ Leptin may be neurotophic and promote neurogenesis,^39^–^42^ and leptin deficient ob/ob mice show reduction of brain size and neuron numbers^43^,^44^ as well as defective glial and synaptic proteins.^45^ Leptin also acts through ObR and mitogen-activated protein kinase (MAPK) to facilitate *N*-methyl-d-aspartate (NMDA) receptor-mediated Ca^2+^ influx.^46^ This suggests a potentially deleterious role of leptin signaling during excess excitation as seen in epilepsy.

Both anti- and proconvulsive roles of leptin have been shown by several groups. The antiepileptic effect of leptin is seen in ob/ob mice, which have increased seizure susceptibility, and in the effectiveness of intranasal leptin to decrease seizures.^47^–^49^ The proepileptic effect is also well characterized. After intracerebroventricular injection, leptin has dose-dependent effects to potentiate penicillin-induced convulsion; lower doses (1 or 2 μg) are most effective, seen 90 min after delivery to the rat, whereas a high dose (10 μg) has no effect.^50^ The proconvulsive effect of leptin appears to be mediated by the NMDA receptor.^51^ The NMDA receptor mediates the leptin-induced excitatory effect by increasing intracellular calcium levels and synaptic transmission in rat hippocampal slices and cell culture.^34^ Nonetheless, the same group of researchers showed that leptin can also act through PI3K and BK channels to inhibit epileptiform-like activity in neurons.^47^ These contradictory findings for leptin may be related to different cellular effects upon activation of astrocytes, microglia, or neurons, as all three cell types express leptin receptors. 22,24

**Figure 2:**
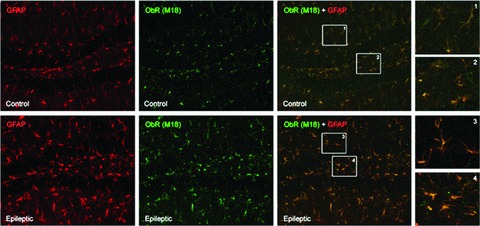
In the hippocampus of a mouse 6 weeks after pilocarpine-induced epilepsy (lower panel), there is increased GFAP (red) and ObR (green) immunoreactivity. Confocal colocalization shows that most ObR is present in astrocytes in control mice (upper panel) and upregulated by seizure.

**Figure 3:**
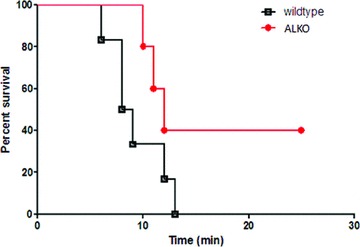
Seizures were induced in female ALKO and littermate controls with a high dose of pilocarpine (350 mg/kg i.p., sevenfold more than the usual dose), 30 min after pretreatment with scopolamine (1 mg/kg i.p.) to reduce peripheral cholinergic effects. The censor time was 25 min. In the control group, all mice died between 6 and 13 min, with a mean survival time of 8.5 min. In the ALKO group, three mice died at 10, 11, and 12 min, and the remaining two mice survived past the censored time of 25 minutes. The hazard ratio is 0.71. The log-rank test shows that the two survival curves had a trend toward significance (*P* = 0.08).

Using a mouse model of epilepsy induced by pilocarpine, we detected robust upregulation of ObR in the reactive astrocytes (Fig. 2). By contrast, neuronal ObR in the hippocampus was only increased with severe seizures, as seen by high Racine scale scores. In the astrocyte-specific leptin receptor knockout (ALKO) mice generated in our laboratory,^52^ high-dose pilocarpine-induced seizures had a milder presentation, and the acute mortality immediately after seizure induction was reduced in comparison with the wildtype littermates (Fig. 3). Better survival and less severe seizures in the ALKO mice in response to a high dose of pilocarpine suggest that astrocytic ObR is detrimental to epilepsy.

With regard to spatial learning and cognitive functions, intrahippocampal leptin administered immediately after training improves subsequent retention of T-maze footshock avoidance and step-down inhibitory avoidance behavior in normal CD1 mice, and has a beneficial effect on memory processing of the SAMP8 mice with their spontaneously accelerated aging and elevated cerebral amyloid β protein.^33^ This is consistent with a dose-dependent facilitatory role of leptin in hippocampal long-term potentiation.^32^ It remains to be determined how astrocytic and neuronal leptin signaling interact with each other in the execution of normal and pathophysiological functions in the hippocampus.

## Astrocytic leptin signaling in the brain regulates the development of obesity

**Figure 4:**
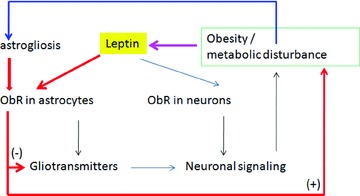
Possibilities of how obesity acts through the astrocytic leptin system to modulate neuronal leptin signaling. Metabolic factors, including leptin itself, may induce reactive astrogliosis and increase astrocytic ObR. Leptin uptake and/or signaling by astrocytes may thus be enhanced and, in turn, affects neuronal leptin signaling.

It is now clear that adult-onset obesity is associated with region-specific upregulation of astrocytic ObR, shown in both agouti viable yellow (A^vy^) mice that have a genetic mutation with constitutive production of a reverse melanocortin receptor antagonist and reduced apparent influx of leptin from blood to the brain,^22^,^53^ and in control C57 mice with adult-onset obesity.^24^ When astrocytic activity is inhibited by pretreatment of the A^vy^ mice with fluorocitrate, intracerebroventricular leptin-induced STAT3 activation is increased in neurons, concurrent with a higher signal intensity of fluorescently conjugated leptin taken up by periventricular neurons.^54^ There are a few possibilities explaining how ObR^+^ astrocytes may affect neuronal leptin signaling: (1) levels of ObR in astrocytes affect leptin permeation across the BBB; (2) once leptin crosses the BBB, it reaches astrocytes first but has a longer diffusion distance to neurons. Astrocytic ObR might compete with neuronal ObR for leptin endocytosis, and (3) astrocytes generate secondary signals in response to leptin, which, in turn, modulates neuronal responses (to leptin and to other stimuli, Fig. 4). Gliovascular coupling (between astrocytes and BBB microvessels) and metabolic coupling (between astrocytes and neurons) are dynamic processes responsible for regulation of cerebral blood flow and metabolism. Leptin is not a vasoactive polypeptide but probably can participate indirectly in these processes.

Astrocytes express several ObR splice variants, including ObRa, ObRb, ObRc, and ObRe. The presence of leptin signaling in astrocytes is shown by an increase of calcium influx in primary astrocytes after leptin superfusion during real-time calcium imaging.^24^ To determine whether the level and isoforms of ObR in astrocytes affect leptin permeation across the BBB, C6 astrocytoma cells were transfected with different ObR isoforms before they were cocultured with hCMEC/D3 cerebral endothelial cells in a Transwell system. The apical-to-basolateral permeation of leptin in the *in vitro* BBB system was unchanged when the C6 cells overexpressed ObRa, but it was increased in C6 cells overexpressing ObRb or ObRe.^55^ This appears to indicate that altered astrocytic leptin signaling facilitates leptin transport across the BBB. However, new preliminary data suggest that ObRa is the major form of upregulated astrocytic leptin receptor. Oppositely, removal of astrocytic leptin signaling does not have a significant effect on baseline transport of leptin across the BBB, since the newly generated ALKO mice do not show a reduction of leptin transport across the BBB in comparison with their wildtype littermates.^52^ Of course, ALKO mice have an embryonic absence of astrocytic leptin receptors, so that compensatory mechanisms could have emerged.

It is yet to be determined whether astrocytic leptin receptor expression or signaling compromise the availability of leptin to ObR^+^ neurons, or whether secondary signals from leptin modulate neuronal leptin activities. This can be achieved by use of a gene knockdown approach before coculture of astrocytes and neurons, or by comparison of astrocytes from ALKO or wild-type mice on their effects on cocultured neurons. Nonetheless, *in vivo* inhibition of astrocytic metabolic activity by fluorocitrate has shown an increase of neuronal uptake of leptin after intracerebroventricular injection, accompanied by an elevation of STAT3 activation.^54^

The border between the area postrema and nucleus tractus solitarius and dorsal vagal complex in the caudal brainstem consists mainly of pallisading astrocytes. The columnar cells compose a continuous monolayer and are immunopositive for both the tight junction protein zona occludin-1 and the astrocyte marker GFAP.^56^ These cells also express ObRa and ObRf as well as ObRb in rats; they not only constitute a diffusion barrier to fluorescent dyes after intravenous or intracerebroventricular injection, but also show upregulation of the ObR short forms.^57^ This reflects regulatory changes of leptin transport from blood to the medulla and perhaps also other parts of the brainstem during energy insufficiency. This important set of data is complementary to the results identifying leptin receptors in selective brain regions in mice with adult-onset obesity,^22^,^24^ and illustrates an important role of ObR^+^ astrocytes in the blood-to-CNS transport of leptin.

**Figure 5:**
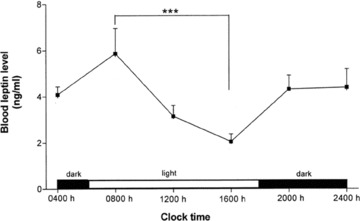
Leptin production and blood concentration show a 24-h rhythm that has a peak in the early morning and nadir in late afternoon in male CD1 mice that also show rhythmicity in blood–spinal cord barrier transport but not blood–brain barrier transport of leptin. Modified from Pan and Kastin.^64^

In the nucleus tractus solitarius, leptin plays an important role in regulating autonomic nervous system activities, including feeding, gastric motility,^58^ and the hypercapnic ventilator response.^59^ Leptin is closely associated with obstructive sleep apnea syndrome,^60^,^61^ whereas continuous positive airway pressure treatment for 8 weeks reduces the concentration of leptin as well as total cholesterol and low-density lipoprotein.^62^ It is interesting to note that obesity attenuates the clock genes *Bmal1* and *Rev-erbalpha* and upregulates peroxisome proliferator-activated receptor alpha in this area.^63^ Consistent with this, there is a circadian rhythm of leptin concentration and leptin transport across the BBB and blood–spinal cord barrier, despite partial saturation (Fig. 5).^64^ This might be associated with a role of leptin in sleep regulation, as leptin-deficient mice have impaired sleep with more arousals and shorter sleep bouts despite an increase in the total amount of nonrapid eye movement sleep.^65^

Astrocytes are well positioned to regulate synaptic transmission and the neurovascular network. In astrocytes, glycogen is metabolized into lactate, which is subsequently transported into neurons to serve as a storehouse for glucose for neurons.^66^ Cholesterol is also transported from astrocytes to neurons where it aids in synaptogenesis.^67^ Astrocytic activation induces the accumulation of arachidonic acid and release of the gliotransmitters glutamate and adenosine-5′-triphosphate (ATP). The role of astrocytes in metabolic coupling has been reviewed extensively.^67^,^68^ In the near future, we shall have a better understanding of how leptin signaling contributes to astrocyte–neuronal communication.

## Summary

Though one of the best known functions of leptin is regulation of feeding behavior, leptin also plays essential roles in the regulation of cerebral blood flow and metabolism, cell differentiation, cognition and learning, and neurodegeneration, where it may play dual roles in stroke or epilepsy. The extrahypothalamic distribution and nonneuronal cellular distribution of leptin form a structural basis for its pleiotropic actions. By illustrating how astrocytic leptin signaling facilitates leptin transport across the *in vitro* BBB and attenuates the development of diet-induced obesity in mice, we have deduced a novel role for the astrocytic leptin system in gliovascular and metabolic coupling. The ObR^+^ astrocytes show dynamic changes in neurological and metabolic disorders, and are intricately linked to CNS functions.
